# Biobanks in the era of personalized medicine: objectives, challenges, and innovation: Overview

**DOI:** 10.1186/s13167-016-0053-7

**Published:** 2016-02-22

**Authors:** Judita Kinkorová

**Affiliations:** 1 Faculty Hospital in Pilsen, Edvarda Benese 1128/13, 305 99 Plzen, Czech Republic; 2 Medical Faculty Charles University in Pilsen, Lidicka 1, 301 00 Plzen, Czech Republic

**Keywords:** Biobanks, Types, History, Definitions, Role of biobanks, Ethical and legal issues, Predictive preventive personalized medicine

## Abstract

Biobanks are an important compound of personalized medicine and strongly support the scientific progress in stratification of population and biomarker discovery and validation due to progress in personalized medicine. Biobanks are an essential tool for new drug discoveries and drug development. Biobanks play an important role in the whole process of patient prevention and prediction, follow-up, and therapy monitoring and optimalization. Biobanks have the specificity in that they cover multidisciplinary approach to the human health combining biological and medical approaches, as well as informative bioinformatics technologies, computationing, and modeling. The importance of biobanks has during the last decade increased in variety and capacity from small collections of samples to large-scale national or international repositories. Collected samples are population-based, disease-specific or rare diseases originating from a diverse profile of individuals. There are various purposes of biobanks, such as diagnostics, pharmacology, or research. Biobanks involve, store, and operate with specific personal information, and as a consequence, such a diversity of biobanking is associated with a broad spectrum of ethical and legal issues. Biobanks are an international phenomenon because any single country, state, or society at the moment is not able to cover all issues involving the whole biobank problematic. Biobanks have an enormous innovative potential in the whole process of biomedical research in the twenty-first century.

## Background

A new era of medical research brought in the last decade a lot of new discoveries, new knowledge, and information and is, by many authors, called the era of personalized medicine. The term covers not only new knowledge and approaches but also new paradigms of current medicine from curing to prevention. It also means a combination of new multidisciplinary approaches to better understand health and diseases. A new era of medical research brings new questions that have to be answered.

**Fig. 1:**
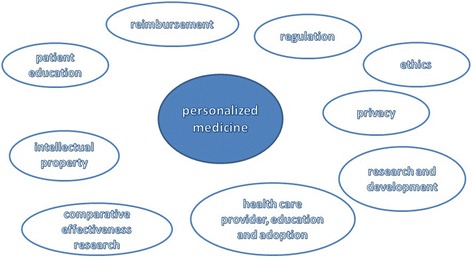
Main impacts of personalized medicine. The implementation of personalized medicine requires a confluence of multiple factors. Full implementation of personalized medicine can only be achieved when all sectors converge toward the center. Modified from [1]

Personalized medicine is as a new approach to the patient built on several pillars: -omics methods (proteomics, metabolomics, and epigenomics), systems medicine, bioinformatics, and biobanks, and the implementation of personalized medicine requires a confluence of multiply factors (Fig. 1) [1].

**Fig. 2:**
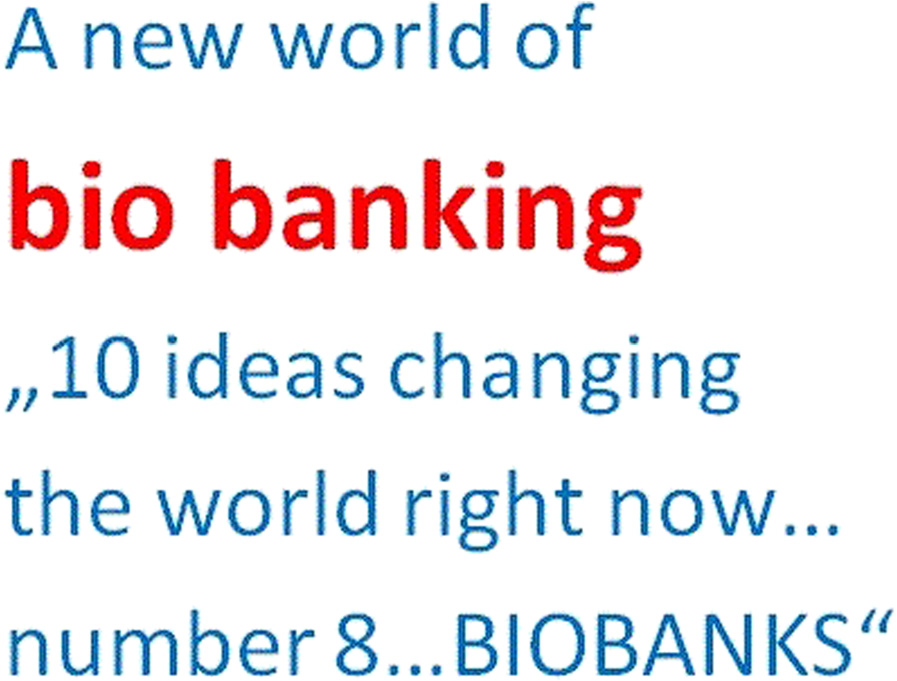
Ten ideas changing the Word right now. In 2009, Time Journal has published ten ideas that had influenced the world’s changes. On the eighth position were biobanks as potential for biomedical research. Modified from [2]

Biobanks are on the list of “10 Ideas Changing the World Right Now,” published in Time 2009 [2]. In this article, the biobank is a safe house for tissue samples, tumor cells, DNA and, yes, even blood—that would be used for research into new treatments for diseases (Fig. 2) [2].

General biobanks are much more flexible as they can support a variety of studies, including cross-sectional studies of genotype-phenotype correlations, case control studies using a biobank for cases and/or controls, and cohort studies using baseline and follow-up data in a biobank to link genetic variation with health outcomes [3].

### Definitions

Biobanks were defined by many authors, institutions, societies, and organizations in many different ways during the last decade.

The big international societies like OECD, ISBER, European Commission, and BBMRI-ERIC have expressed their definitions.

The Organisation for Economic Co-Operation and Development (OECD) defines a biobank as a collection of biological material and the associated data and information stored in an organized system, for a population or a large subset of a population [4].

OECD in Recommendation on Human Biobanks and Genetic Research Databases (HBGRD) [5] provides guidance for the establishment, governance, management, operation, access, use, and discontinuation of human biobanks and genetic research databases, which are structured resources that can be use for the purpose of genetic research and which include:

- Human biological materials and/or information generated from the analysis of the same;
- Extends it with associated information.

International Society for Biological and Environmental Repositories (ISBER) defines biobank as an entity that receives, stores, processes, and/or disseminates specimen as needed. It encompasses the physical location as well as the full range of activities associated with its operation [6].

Also, European Commission (EC) gave a definition. Joint Research Centre (JRC) published in 2015 in Scientific and Technical Reports a paper, Biobanking in Europe: prospects for Harmonization and Networking, which gives the following definition: biobanks are organized collections consisting of biological samples and associated data of great significance for research and personalized medicine [7]. Two years later, the European Commission published another document: Report of an expert group on Dealing with Ethical and Regulatory Challenges of International Biobank Research: Biobanks for Europe, A challenge for governance with more comprehensive definition. Biobanks typically:

- Collect and store biological materials that are annotated not only with medical, but often also epidemiological data;
- Are not static “projects,” since biological materials and data are usually collected on a continuous or long-term basis;
- Are associated with current and/or future research projects at the time of specimen collection;
- Apply coding or anonymization to assure donor privacy but have, under specific conditions, provisions that participant remain re-identifiable in order to provide clinically relevant information back to the donor;
- Include established governance structures and procedures (e.g., consent) that serve to protect donors’ rights and stakeholder interests.

At the moment, the biggest player in the field of biobanks, Pan-European Biobanking and Biomolecular Resources Research Infrastructure (BBMRI), defines biobanks as follows: biobanks contain biological samples and associated information that are essential raw materials for the advancement of biotechnology, human health, and research and development in life science [8].

Also, many authors published various definitions of biobanks with respect to one or some special features of biobanks.

The very simple but generally accepted definition was published by Kauffmann and Cambon-Thomsen in 2008 [9]: biobank is an organized collection of human biological material and associated information stored for one or more research purposes.

And finally, the definition offered by Artene et al. [10] clearly describes the biobank as a set consisting of two different parts:

- The biological material that is collected, processed, and long-time stored;
- The database, including information about demographical and clinical data for each sample and also associated with the bank inventory with the main activities: biospecimen collection, processing, storage or inventory, and distribution of biological material.

Generally, the definition of biobank consists of three groups of relatively distinct information:

- Biological human material;
- Attached or connected information;
- The legal issues like consent and patient/individual data safety and protection.

It makes the science of biobank so comprehensive and complicated because it requires a multidisciplinary and universal approach in all stages of life cycle of the biobank.

### Time for biobanks

The idea of biobanks is not new. In many countries, biological samples not only of human origin were collected more or less systematically for many decades, with more or less connected information of the individual. These samples were taken randomly, collected, stored, shared, and provided for other purposes in the beginning without regulations and rules. At the time the requirements for the type of the sample (blood, urine, RNA, DNA, tissue …) size and the way how the sample is taken, transported, saved, and preserved became more specific and precise, it meant the quantitative and qualitative characteristics of samples were specific according to the advances in biomedical and clinical trial or research. Such great event like the sequencing of human genome in 2001 opened the door to new methods on how to study diseases and disorders. New and emerging technologies are based on improved molecular profiling, better understanding of factors, and processes leading to understanding of the ethiopathology of diseases. These achievements enabled new approach to the health care based on individual genomic, proteomic, and metabolomic profiles [11]. The side effect of the progress in biomedical research is the emergence of “big data,” large-scale data, and information about patients’ characteristics, diseases, and epidemiological, environmental, lifestyle, and societal data which require a new approach to handle the whole process of the sample manipulation, and consequently biobanks.

**Fig. 3:**
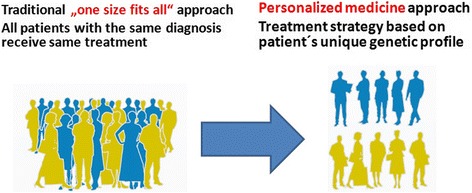
Personalized medicine contribution to better health care. Stratification of patients is another result and requirement for the biobanks that enable shift from “one-size-fits-all” 11 to more targeted therapy, therapy models, and in silico therapies. A stratified approach enables to improve the individual health care, with respect to age, sex, demography, and relevant costs. Modified from [12]

Stratification of patients is another result and a requirement for biobanks that enable a shift from “one-size-fits-all” [11] to more targeted therapy, therapy models, and in silico therapies. A stratified approach based on detailed information about the individual biological variation, complemented with environmental, life factors, and societal information enable to study the health and diseases in complexity and to improve the individual health care, with respect to age, sex, demography, and relevant costs (Fig. 3) [12].

New knowledge in informatics, bioinformatics, and information technologies strongly supported and still supports the formation and development of biobanks.

Biobanking has been identified as a key area for development in order to accelerate the discovery and development of new drugs, recently especially in oncology [13].

Biobanks play an important role in identification of new biomarkers. Population surveys and biobanking research are essentials tools in the elucidation of the etiology of complex diseases and the molecular basis of disease subtypes. A more precise biology-based classification of disease speeds up the development of highly sensitive, high-throughput methods more targeted, effective, and cost-effective treatment, reduces the incidence of undesired side effects of therapy, improves access in clinical and pharmacology trial design, and leads to new concepts of disease prevention and health promotion [14]. Zatloukal and Hainaut [15] suggest a novel way of structuring biobank network according to the clinical trial design, thus creating a step-by-step process, starting with assessing and measuring identified biomarkers, through to whether the biomarker is informative in determining the trialed drug’s effect.

### New dictionary for biobanks

As biobanking is developing to the science of biobanking, new requirements are appearing regarding definitions, obligations, and terminology. Scientists from different scientific branches do not understand the terms in the same way, and even between countries, significant differences can be found. Fransson in his study [16] clarifies the general understanding of several most used terms: biobank, sample, specimen, aliquot, coding, anonymizing, personal data, and patient’s informed consent. Some new terms have to be defined or redefined for the purposes of biobanks like genetic data, biometric data, and impact assessment. The study shows a considerable confusion in some of the terms used in the biobank community.

### Modern history of biobanks

Biospecimens have been collected and stored in conjunction with clinical and epidemiological studies for several decades. The first biorepositories have existed in various forms for over 150 years, from early small collections to modern automated facilities managing millions of samples [17].

Human specimens have been collected and stored at institutions in the USA and elsewhere for over 100 years. Historically, these stored tissue samples have been used by the biomedical community for educational and research purposes. More recently, the stored tissues have played a major role in understanding and treatment of diseases [18]. The history of real biobanks is not long, about 30 years. The first banks were repositories of randomly collected samples and information; data associated with stored biospecimens have increased in the mean time in complexity from basics, such as date of collection and the diagnosis, to extensive information sets encompassing many aspects of participant or patient phenotype, now rapidly extending into genetic, proteomic, and other -omics information. From the historical point of view, the chronologic development was followed by De Souza and Greenspan [19]:

- Academic/university-based repositories, possibly the first biobanks in existence, developed almost exclusively around single-project goals and research requirement;
- Institutional- or government-based biobanks that hold greater numbers of samples for a wider research purposes;
- Commercial/for-profit biorepositories;
- Population-based biobanks, where long-term sample acquisition from broad populations enables longitudinal studies such as disease monitoring, aging studies, and biomarker discovery;
- Virtual biobanks that hold no physical specimens but offer location and retrieval services for samples held globally or nationally.

Because the science of biobanking is very closely linked to the development of an enabling infrastructure, it requires scientists to work more closely with each other and with funders than has historically been the norm in biomedical science [20].

### Types of biobanks

Before the biobanks classification from various sources will be presented, the difference between biobank and biorepository has to be clarified: the term biobank has been used interchangeably with biorepository [21]. Previously OECD used the term “biological resource center” not only for repositories, but also for suppliers of health research services [5]. The International Agency for Research on Cancer (IARC) used the term “biological resource center” for collections of human cancer samples [22]. In the USA, the National Cancer Institute defined the term biorepository as an organization, place, room, or container where biospecimens are stored…in a freezer by an individual researcher [23]. Likewise, the term biobank has been used in this context by other US and European institutions [22].

Classification of biobanks is not simple and is based on different approaches: there are various types of biobanks like population-based, diseases-oriented, hospital- or academic- based, networked, or run by the government, non-profit organizations, or commercial companies among others [24].

Biobanks also vary in scale, nature, contents, and participants. Some definitions overlap, and in definition of biobanks, they may be “radically contrasting views over how certain attributes should be identified, formulated, defined, or ranked…” [25].

Human biobank classification (according to biomedinvo4all website) [26] is based on:

- Tissue type (tumor tissue, cells, blood, DNA, or DNA array results);
- Purpose/intended use (research, forensics, transplantation, source for therapeutics, e.g., umbilical blood, stem cell biobanks for individual or community use, or diagnostics);
- Ownership (academic and research institutions, hospitals, biotechnology and pharmaceutical companies, and stand-alone biobank companies and foundations may hold biobanks.

Ownership may be private, public, or in partnership across sector boundaries (ownership may be public, managed in partnership with government).

- Volunteer group/group of participants (population-based, such as all newborns, adults, or pregnant women, or disease-based, including only those with a specific disease).
- Size (disease group, regional, statewide, or national).

Gottweis and Zatloukal [27] defined four basic types of research biobanks:

- Clinical/control based on biological specimen from patients with specific diseases and from non-diseased control;
- Longitudinal population-based biobanks that follow a portion of the population over a large period of time;
- Population isolate biobanks with a homogenous genetic and environmental setup of the population represented;
- Twin registries with samples from monozygotic and dizygotic twins.

Rebulla et al. [28] classification is even broader and identifies six types of biobanks:

- Leftover tissue biobanks collected during clinical pathology diagnostic procedures;
- Population biobanks;
- Twin biobanks;
- Disease biobanks from patients suffering from a specific condition;
- Organ biobanks;
- Nonhuman biobanks.

Currently, the generally accepted classification comes from the pan-European Biobanking and Biomolecular Resources Research Infrastructure (BBMRI) [8], which distinguishes between only two types of biobanks:

- Population-based biobanks (population-based prospective biobanks focused on the study of the development of common, complex diseases over time).
- Disease-oriented biobanks (biobanks of tissue samples and clinical data also referred to as disease oriented or clinical biobanks).

### What kind of human material is collected in biobanks?

The world’s most comprehensive directory of biobanks, tissue banks, and biorepositories collect blood, whole blood, plasma, serum, RBC, white cells, ducal swab, DNA, RNA, protein, cell lines, fluid, urine cerebrospinal fluid, synovial fluid, amniotic fluid buffy coat, bone marrow stem cells, and tissue provided by Global Bank Directory, Tissue Banks, and Biorepositories [29].

### Infrastructures and international projects of biobanks

Biobanks encompass a unique research infrastructure that requires different governance mechanisms than project-based research. The governance mechanisms must balance the needs of the scientific community and the participants with an emphasis on the recognition of participants, trustworthiness, and adaptive management [30].

In recent years, biobanks across the globe have received much attention as a new key infrastructure and resource for biomedical research and drug development. Increasingly, biobanks are becoming networked and even international projects in the context of post-genomic medical research [31].

Biobank consortium EuroBioBank (EBB) network (www.eurobiobank.org) was the first operating network of biobanks in Europe to provide human DNA, cell, and tissue samples for research on rare diseases (RDs). The EBB was established in 2001 to facilitate access to rare disease biospecimens and associated data; it obtained funding from the European Commission in 2002 as a project in the *5th Framework Programme* (FP5) and started operation in 2003 [32].

The most important and the biggest infrastructure in Europe and even all over the world is Biobanking and BioMolecular resources Research Infrastructure—European Research Infrastructure Consortium (BBMRI-ERIC, http://www.bbmri-eric.eu/). BBMRI:

- Pan-European distributed infrastructure of existing and new bio-banks and biomolecular resource centers;
- Provides access to human biological samples that are considered as essential raw material for the advancement of biotechnology, human health, and research and development in Life Sciences (e.g., blood, tissues, cells or DNA that are associated with clinical and research data);
- Comprises biomolecular research tools and bio-computational tools to optimally exploit this resource for global biomedical research.

The mission of BBMRI-ERIC is to increase efficacy and excellence of European biomedical research by facilitation access to quality-defined human health/disease-relevant biological resourced through:

- The inclusion of associated data in an efficient and ethically and legally compliant manner;
- By reducing the fragmentation of the biomedical research landscape through harmonization of procedures, implementation of common standards, and fostering high-level collaboration;
- By capacity-building in countries with less developed biobanking communities thereby contributing to Europe’s cohesion policy and strengthening the ERA.

### The science of biobanks

The science of biobanks is very broad and diverse and includes research, education, funding, publishing, biobanking services, and others. A lot of activities have appeared to support the development of biobanks. In 2005, Office of Biobanking and Biospecimen Research (OBBR) was based in the frame of US National Cancer Institute (NCI). Also, in Europe, a lot of activities supporting the biobank development have been raised, some of them in the frame of *7th Framework Programme* of EU (FP7) in years 2007–2013 and continues in the following framework program, Horizon 2020 (2014–2020). EU funded projects are pioneering the development of techniques for population genetics and performing large population studies on the genetic predisposition to major diseases. Support is also provided for development of harmonization protocols and for collection, storage, and management of patient samples and of genetic data across Europe. Recognizing the power of population-based approaches to study genetic susceptibility for disease, between 2002 and 2008, the European Commission’s Framework Programmes for Research and Technology Development (RTD) have provided more than €60 million to collaborative research projects in this area. The most relevant projects are mentioned here.

Project P3G (the Public Population Project) in Genomics is an international consortium with members in 40 countries. It aims to lead, catalyze, and coordinate international efforts and expertise, so as to optimize the use of studies, biobanks, research databases, and other similar health and social research infrastructures (http://www.p3g.org).

Project SPIDIA (Standardisation and improvement of genetic Pre-analytic tools and procedures for In-vitro DIAgnostics, http://www.spidia.eu) was launched in 2009 and brought together 16 academic institutions, international organizations, and life sciences companies. The project aid is to standardize and improve pre-analytical procedures for in vitro diagnostic testing [13].

The ENGAGE (http://www.euengage.org/) consortium has brought together 24 leading research organizations and two biotechnology and pharmaceutical companies across Europe and in Canada and Australia. ENGAGE aims to translate the wealth of data emerging from large-scale research in genetic and genomic epidemiology from European (and other) population cohorts into information relevant to future clinical applications. The concept of ENGAGE is to enable European researchers to identify large numbers of novel susceptibility genes that influence metabolic, behavioral, and cardiovascular traits and to study the interactions between genes and lifestyle biomarkers factors.

The ENGAGE consortium will integrate and analyze one of the largest ever human genetics dataset (more than 80,000 genome-wide association scans and DNAs and serum/plasma samples from over 600,000 individuals). One goal is to demonstrate that the findings from ENGAGE can be used as new diagnostic indicators for common diseases that will help us to understand better risk factors, disease progression, and why people differ in responses to treatment.

HYPERGENES project (http://www.hypergenes.eu) is focused on the definition of a comprehensive genetic epidemiological model of complex traits like essential hypertension (EH) and intermediate phenotypes of hypertension dependent/associated target organ damages (TOD) as well as other endophenotypes as the pharmacogenomic pattern of drugs widely used in EH. The discovery of the genetic component in common complex diseases is extremely challenging since most of them are multifactorial and since the genetic component is likely to be described by the interactions of several genes involved in the disease pathway, each predisposing imperceptibly to the disease. HYPERGENES adopted the genome-wide association (GWA) approach to identify common variants contributing to the inherited component of common diseases.

The GEN2PHEN project (http://www.gen2phen.org/) aims to unify human and model organism genetic variation databases toward increasingly holistic views into genotype-to-phenotype (G2P) data, and to link this system into other biomedical knowledge sources via genome browser functionality. The project will establish the technological building-blocks needed for the evolution of today’s diverse G2P databases into a future seamless G2P biomedical knowledge environment, by the project’s end. This will consist of a European-centered but globally networked hierarchy of bioinformatics GRID-linked databases, tools and standards, all tied into the Ensemble genome browser.

All together, 34 projects were/are supported by European Commission in FP7, and the process continues in Horizon 2020.

**Fig. 4:**
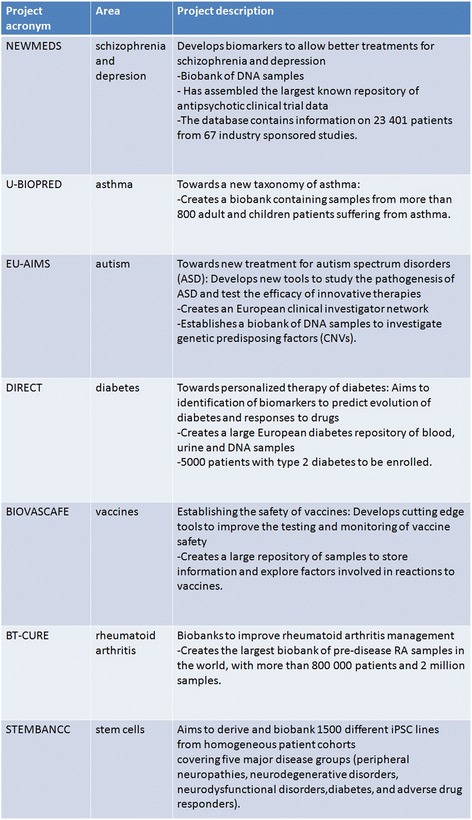
The projects including biobanking activities supported by Innovative Medicines Initiative 2 (2009–2014). Modified from [32]

Not only framework programs of European Union support the biobanking in Europe and all over the world, but also other international initiatives contribute to the development of biobanking. Innovative Medicines Initiative (IMI, http://www.imi.europa.eu/), Europe’s largest public-private initiative, is aiming to speed up the development of better and safer medicines for patients. IMI supports collaborative research projects and builds networks of industrial and academic experts in order to boost pharmaceutical innovation in Europe. IMI is a joint undertaking between the European Union and the pharmaceutical industry association—European Federation of Pharmaceutical Industries and Associations (EFPIA) (Fig. 4) [33].

### Internalization

A multitude of national and regional population-based and disease-oriented biobanks have been established in Europe. However, the exchange of data and materials within national legal frameworks is still difficult and European biobanking efforts are characterized by fragmentation [34]. Despite the unique European strength, value and irreplaceable national collections suffer from underutilization due to fragmentation of the European biobanking research community. Promising international initiatives are challenged by the heterogeneous legal, ethical, and societal environments. There is an urgent need for coordination and harmonization of the biobanking.

Many of the scientific institutions, which are currently in the process of establishing or using biobanks may be operating in a legal “gray zone,” because:

- There are currently very few specific legal regulations pertaining to such collections;
- Where such regulations do exist, they vary greatly between different countries;
- Solid experience of legal practice is widely lacking in the field of biobanking.

Furthermore, a comprehensive assessment of the legal standing of a biobank would severely strain the logistical and financial resources of most interested institutions [35].

Biobanks are embedded in complex networks of research collaborations that span regions and countries. At the moment, no single laboratory, institution, or country is able to cover the whole problematics of biobanking. International multidisciplinary cooperation is the cornerstone in the complex process of biobank development and operational functioning.

### Biobanks, bioinformatics, and ICT

The ability to correlate data and biospecimens from different biobanks is crucial to accelerating the pace of translational research. A meta-model to describe information about a biobank is already under construction as a first-step data sharing among biobanks that exhibit tremendous heterogeneities. This work is being conducted internationally to help harmonize the national biobanks participating in the Biobanking and Biomolecular Resources Research Infrastructures Initiative (BBMRI) (http://www.bbmri.eu/index.php/). Information about the participating biobanks is captured by a common set of attributes (minimum data set) designed to adopt different kinds of collections. The latest interoperability and semantic web technologies can be used for building resource description frameworks for data and services providing flexible frameworks that can be used in different data-sharing scenarios.

### Ethical, legal, and social issues (ELSI)

Ethical issues are commonly present in many aspects of biobanking. The fact that biobanks deal with human samples, invading an individual autonomy or limiting self-control, provokes a number of ethical issues [36].

The use of biobanks is increasing and raises a lot of ethical, legal, and social issues. In addition to these, there are also other issues which have to be taken in account such as equipment which means processing, annotation, storage, and operating procedures like samples accrual, processing, annotation, storage, release, distribution, and tracking. Any kind of related information must be considered: clinical informatics like pathology, treatment, outcome data, and database structures and as mentioned above, patient (informed) consent, preference list, inventory management tools, and query tools. And finally, national policies, economic models including funding sources, user fees, intellectual property, governance models, and education and training of all stakeholders (the donors, investigators, funding agencies, institution housing the biosamples and ethics review committee) have to be included.

Currently, most actual questions that have to be answered are as follows. What are the ethical trends and legal frameworks in the post-genomic era? Are there new issues in relation to the developments of techniques and new study designs? How does this affect the clinician’s attitudes and relationship with the patients? The main ethical issues encountered are informed consent, confidentiality, secondary use of samples and data over time, and return of results [37, 38].

### Informed consent

Informed consent [39] according to international conventions and guidelines principles in research ethics is to guarantee the voluntary participation in research and address privacy issues in research. Informed consent consists of three basic components: adequate information, voluntariness, and competence. It means that prior to consenting, a participant should be informed of the goal of his participation and research, possible risks and adverse event, and the possibility to refuse or withdraw from research at any time. Informed consent is required when the research involves the participation of human beings, when the research uses genetic material or biological samples, and when the research involves personal data. Informed consent should respect individuals’ autonomy and vulnerability. Special attention must be paid to specific groups of participants like children, elderly, mentally deficient persons, severely injured patients, and participants with specific cultural or traditional background, ethnic specificity, and so on.

Recently, the discussion has focused on the problem if the consent has to be general or broad [37]. The more general consent is less informed; on the other hand, it averts all aspects relevant to the patient’s choice. The existence of different terms has posed a major problem for discussions on confidentiality issues. European Medicines Authority (EMEA) has proposed the terminology and nomenclature that has been adopted by the International Conference on Harmonization of Technical Requirements [40]. The nomenclature is in basic terms as follows: identified data and samples are labeled with personal identifiers such as name or identification numbers; coded data and samples are labeled with at least one specific code and do not carry any personal identifiers; and anonymized data and samples are initially single or double coded, but the link between the subjects’ identifiers and the unique code(s) is subsequently deleted. Once the link has been deleted, it is no longer possible to trace the data and samples back to individual subjects through the coding key(s). The discussion about absolute safety has led to the new term open consent model proposed by Lunshof et al. [41], which refrains from any promises of anonymity, privacy, or confidentiality [31].

### Confidentiality, privacy, and data protection

Data protection and privacy are fundamental human rights which need to be protected at all time [42]. Participants want to have control over their personal information and personal communications, and these should be treated confidentially. Data protection guarantees the right to privacy. Data protection reveres to the technical framework and security measures designed to guarantee that all personal data are safe from unforeseen, unintended, or malevolent use. Data protection therefore includes both measure with regards to access to data and the conservation of data. Also, accuracy of data can be included in a data protection strategy.

A very important issue is (personal) data ownership. Since there are clearly multiple stakeholders in a biobank—the donors, investigators, funding agencies, institution housing the biosamples, and ethics review committee—it has been proposed that the institution of the biobank should hold “custodianship” for the use of the resource, and that the custodian of the samples should fulfill numerous responsibilities [43].

Sharing the best practices and procedures in biobanks requires the process of harmonization. Harmonization is a more flexible approach aimed at ensuring the effective interchange of valid information and samples [44]. The importance of the harmonization process is to articulate those situations in which true standardization is required. Standardization means precisely the same protocols/standard operating procedures (SOPs) used by all biobanks. Likewise, comparison of high-throughput-technology-derived data requires that platforms and operational details be identical. Harmonization is context-specific and pertains to the compatibility of methodologies and approaches to facilitate synergistic work. It thereby relates to the critical areas of generating, sharing, pooling, and analyzing data and biological samples to allow combining resources and comparing results obtained from different biobanks. Harmonization includes technologies and procedures for phenotype characterization, sample handling, in vitro assays, computational biology analytical tools and algorithms, data-coding, and electronic-communication protocols that enable biobanks to network together within compatible ethic-legal frameworks [20].

Harmonization initiatives have brought together individuals with diverse expertise. On the basis of consensus, they have developed standards, tools, technologies, and resources, which are widely available to the biobanking community today [45]. Currently, the biobanks differ in structure, purpose, and design. That is why they contribute to the generation and translation of knowledge to clinics, public health, and technology in different ways, and the process of harmonization of the practices, polices, and operations is heavy going. Despite this, harmonization fosters the amount of data and specimens useable, and translational science will rely on fundamental biological data to (re)classify human disease on the basis of causality and to identify relevant drug targets and biomarkers [46].

### Biobanks and personalized medicine

Personalized medicine is currently characterized as “4P,” personalized, preventive, predictive, and participatory [47]. Biobanks can contribute to all of these characteristics:

- Personalization reflects the achievements in genetic science in individualized digital genome.
- Predictiveness reflects the ability to predict the risk of certain disease based on information of individual genome in combination with additional information like age, sex, lifestyle, and social and environmental data.
- Preventiveness reflects the individual ability to avoid or minimize risk factors for certain disease.
- Participation reflects the individual proactive behavior in the whole process of health care; it means empowerment of individuals to undertake informed decisions about their health future.

The European Association for Predictive, Preventive & Personalised Medicine (EPMA) as a key player in the field of personalized medicine in Europe consider biobanking as an integral part of personalized medicine approach to the health care and strongly supports the future biobank development [48–50].

### Future of biobanks

**Fig. 5:**
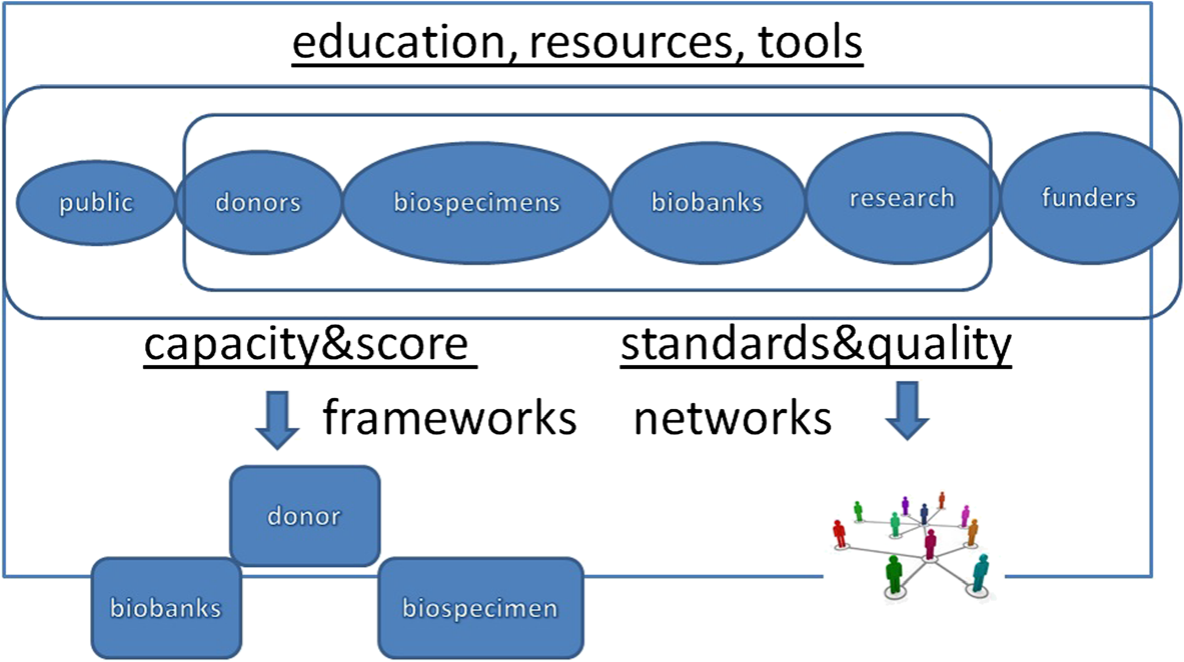
The process of biobanking. Physicians and health care staff members fulfill essential roles in biobanking, which frequently intersects with routine clinical activity. By obtaining specific biobanking knowledge and expertise, individuals will be uniquely positioned to play leadership roles in this cross-cutting field within their institution. Modified from [52]

Despite the progress, biobanks still face a lot of issues to be solved in the whole process of biobanking (Fig. 5) [51]. Biobanking is facing the lack of harmonization, lack of standards, agreed vocabulary, common data elements, and best practices for collecting data and processing samples. An accreditation and evaluation system to recognize biobanks that provide high-quality samples, and reward and acknowledge scientists, who establish and maintain biobanks, should be established [32].

However, most European citizens have never heard of biobanks, nor do they know of their importance in research. Education at any level is the underestimated issue. The current discussion is on people’s and/or patients’ engagement with biobanks and people’s willingness to participate in biobanks.

Furthermore, the legal and regulatory frameworks that apply to this area are fragmented little interest in funding small biobanks that contain and exchange limited numbers of samples.

Long-term sustainability is a major challenge for biobanking. A number of considerations are critical to keeping biobanks and the research they support active and dynamic. The need for long-term investments into biological resources is clearly evident when longitudinal data are needed. Prospective collections of data and samples from asymptomatic individuals will allow future identification of premorbid and subclinical periods of disease development and will identify prognostic and diagnostic biomarkers and drug targets [52].

## Conclusions

Biobanks are complex systems of systematically programmed storage of human material and associated data. In the past 20 years the science of biobanks has became an integral part of personalized medicine and a great number of biobanks have been established all over the world to support the dramatic development in diseases prevention, prediction, diagnosis and treatment.
